# Experimental and Computational Studies on Non-Covalent Imprinted Microspheres as Recognition System for Nicotinamide Molecules

**DOI:** 10.3390/molecules14072632

**Published:** 2009-07-17

**Authors:** Roberta Del Sole, Maria Rosaria Lazzoi, Mario Arnone, Fabio Della Sala, Donato Cannoletta, Giuseppe Vasapollo

**Affiliations:** 1 Dipartimento di Ingegneria dell’Innovazione, Università del Salento, via per Monteroni, km1, 73100, Lecce, Italy; 2 National Nanotechnology Laboratory of CNR-INFM, Distretto Tecnologico ISUFI, Università del Salento, Via per Arnesano, Km 1, 73100 Lecce, Italy

**Keywords:** molecular imprinting, UV-vis spectroscopy, NMR, nicotinamide (nia), theoretical DFT studies

## Abstract

Molecularly imprinted microspheres obtained by precipitation polymerization using nicotinamide (nia) as template have been prepared and characterised by SEM. How various experimental parameters can affect microsphere morphology, reaction yield and re-binding capacity have been evaluated. Pre-polymerization interactions between template and functional monomer in chloroform and MeCN have been studied by ^1^H-NMR. The results suggest that the interaction between nia and methacrylic acid (MAA) is mainly based on hydrogen-bonding between amide protons and MAA. Computational density functional theory (DFT) studies on MAA-nia complexes have been also performed to better understand hydrogen-bonding interactions. The imprinted activity of the microspheres, synthesized in chloroform or acetonitrile (MeCN), has been evaluated by spectrophotometric analysis of nia solutions when chloroform or MeCN are used as incubation solvents. The results suggest that MeCN interferes with hydrogen bonding between template and MAA during either the polymerization step or re-binding process as also observed from theoretical results. Finally, the selectivity towards selected nia analogues has been also confirmed.

## 1. Introduction

Molecularly imprinted polymers (MIPs) are synthetic polymers capable of selectively recognizing a specific target molecule (template). The template, added during the polymerization process, is removed by washing, leaving selective binding sites in the polymer network. The polymer obtained can exhibit in this way high affinity towards the template molecule which can be selectively re-bound to the specific sites. Nowadays MIPs are considered a convenient approach for the development of molecular recognition systems and have important applications in various areas, such as solid-phase extraction [1,2,3,4], chromatography [5,6,7], assays and sensors [8,9,10], catalysis [11,12], chemical traps and drug delivery systems (DDSs) [13,14] even if MIPs- based DDS are yet at an early stage.

Templates interact specifically with functional monomers in different way to make covalent [15,16], non-covalent [17,18] or semi-covalent complexes. In this study non-covalent method has been chosen since it is considered the most straightforward and flexible one even if the weak interactions involved in the complexes formation, generally hydrogen-bonding, electrostatic or π-π interactions, may generate heterogeneous binding sites.

Besides traditional bulk polymerization, other procedures have been developed in order to improve control of MIP particles morphology, such as suspension polymerization [19], multi-step swelling polymerization, sol-gel imprinting [20] and precipitation polymerization [21,22]. Our efforts to obtain homogeneously sized MIP particles led us to use a precipitation polymerization method which provides homogeneous microspheres [23,24,25]. This technique is an economical and labour-saving method and does not require addition of surfactants or stabilizers. In the precipitation technique a diluted system, obtained by using a high amount of a porogenic solvent, is needed in order to obtain a dispersion of micro-gel particles during the polymer synthesis; then the polymer microspheres are easily recovered by washing and centrifugation operations.

In our work nicotinamide (nia), which is an important molecule widely diffused in nature as a form of vitamin B_3_, has been selected as target molecule; nia is part of the nicotinamide adenine dinucleotide (NAD) and nicotinamide adenine dinucleotide phosphate (NADP) coenzymes involved in various redox processes in the human body. It is both a food nutrient and a drug used in pellagra therapy and in the treatment of some neurodegenerative diseases [26]. Moreover, during the last few years its utilization in different health branches is receiving increasing attention. To our knowledge, only a few papers on nia-MIP systems have been reported. Fu and coworkers [27] prepared macro-porous monolith imprinted polymers for nia and its positional isomers using bulk polymerization and their application in the separation of the isomers by using MIP particles as a HPLC stationary phase was also discussed. Wu and coworkers [28] proposed a computational model to simulate the synthesis of bulk MIPs, the removal of template and the recognition of the template and of its analogs. They have calculated the interaction energy between the monomer and the template and they used their computational model in prediction of chromatographic behaviour. Successively, in another paper Wu and coworkers [29] used the same theoretical model focusing on the prediction of solvent effect on recognition properties, which was neglected in their previous work. They found that the small dielectric constant and aprotic solvent were likely to lead to a large interaction energy between template and monomer. Li and coworkers [30] reported the isotherms of nicotinamide and nicotinic acid obtained using nia-imprinted polymer as stationary phase. Zhang and coworkers [31] prepared piezoelectric sensors modified with MIPs synthesized by bulk polymerization, investigating also the response time of the sensors. To our knowledge, there are no studies of nia-MAA pre-polymerization interactions, no examples of MIP-nia systems obtained by precipitation polymerization and no studies of some critical experimental parameters in polymer synthesis procedure.

Since the complexation of the nia template with the functional monomer MAA involves non-covalent hydrogen bond interactions the choice of an appropriate method for the theoretical description of these systems is challenging [32]. Normal Hartree-Fock (HF) computations that do not include electron correlations are not suitable for this purpose. For high level post-HF methods like coupled clusters [CCSD(T)], the studied systems are too big to have an efficient theoretical description. An alternative to such methods lies in density functional theory (DFT) which due to the nature of the employed functionals includes some parts of the electron correlation [33,34]. Especially in combination with the resolution of identity (RI) approximation a very efficient theoretical description of bigger molecular systems is possible [35,36]. The currently available DFT methods work well for the structural and energetic description of hydrogen bond systems [32,37,38,39,40]. This holds especially for the widely applied gradient corrected functionals like BLYP [41,42], B3LYP [42,43], or the PBE [44,45] functional [46,47]. Another important point in the theoretical description of hydrogen bonds is the choice of the basis set and the resulting basis set superposition error (BSSE). This error is a purely mathematical artefact that can be eliminated by the counterpoise (CP) correction that was introduced by Boys and Bernardi [48]. Although the BSSE is found to be quite big for the energies of small hydrogen bonded clusters [49,50] for larger systems that contain H-bonds as structural feature its value decreases and becomes less basis set dependent [47,51].

In a previous work we prepared polymeric microspheres by using a non-covalent imprinting technique and precipitation polymerization of methacrylic acid (MAA) in the presence of 1,8-diaza-bicyclo[5.4.0]undec-7-ene (DBU) as the template [52]. Now, in continuation of our interest in MIP research, we report herein a study of a molecular imprinted system based on non-covalent interactions between nia template molecules and MAA functional monomers. At the same time pre-polymerization studies on the possible interactions between template and functional monomer have been performed by ^1^H-NMR and computational density functional theory (DFT) studies.

A similar approach was successfully used by Prachayasittikul and coworkers for the evaluation of MIPs prepared by a non-covalent method [53,54], even if various differences can be observed in comparison with our work such as template, functional monomers, computational approach and so on. The author found that computer simulations confirmed the hypothesis derived from the experimental data.

The preparation of imprinted microspheres by precipitation polymerization of functional monomer/cross-linker, methyl methacrylic acid/ethylene glycol dimethylacrylate (MAA/EGDMA) in the presence of nia as print molecule has been achieved and some experiments have been performed in order to evaluate how various parameters can affect microsphere morphology, reaction yield, re-binding capacity and so on. Polymeric microspheres obtained in this way have been characterised by SEM studies and binding ability of MIP systems towards different concentration of nia solutions in various solvent systems has been evaluated by spectrophotometric analysis. As a control, binding capacity of nia imprinted poly-(MAA-EGDMA) and non-imprinted poly-(MAA-EGDMA) have been compared. Finally, the selectivity towards nia analogues, such as nicotine and its isomers isonicotinamide (isn) and picolinamide, has been also discussed.

## 2. Results and Discussion

### 2.1. Polymer Syntheses and Characterization

MIPs performance depends on many different parameters such as cross-linker/functional monomer ratio, temperature, type and concentration of monomers and solvent. In this work we have studied the influence of some of these parameters in order to optimise the experimental conditions. The polymers have been characterized by SEM and their average microsphere diameters, the yield of all polymers, and their experimental conditions are reported in Table 1.

**Table 1:** Experimental conditions of MIP and NIP synthesis ^a^, yields and microsphere size.

Sample	Cross-linker EGDMA (mmol)	Solvent	Solvent Volume (mL)	T (°C)	Reaction Time (h)	Yield (%)	Microsphere size (μm)
**P1**	6.19	CHCl_3_	40	60	20	59	(2.0±0.2)
**N-P1**	6.19	CHCl_3_	40	60	20	62	(2.0±0.2)
**P2**	6.19	MeCN	40	60	20	<5	-
**P3**	6.19	MeCN	40	65	20	19	(0.20±0.02)
**P4**	6.19	MeCN	30	65	20	22	(0.35±0.02)
**P5**	6.19	MeCN	30	70	20	43	(0.38±0.02)
**P6**	6.19	MeCN	30	70	48	45	(0.34±0.02)
**P7**	9.3	MeCN	30	70	48	98	(0.50±0.05)
**N-P7**	9.3	MeCN	30	70	48	95	(0.50±0.05)

^a^ 1.55 mmol of functional monomer (MAA) were used.

**Figure 1:**
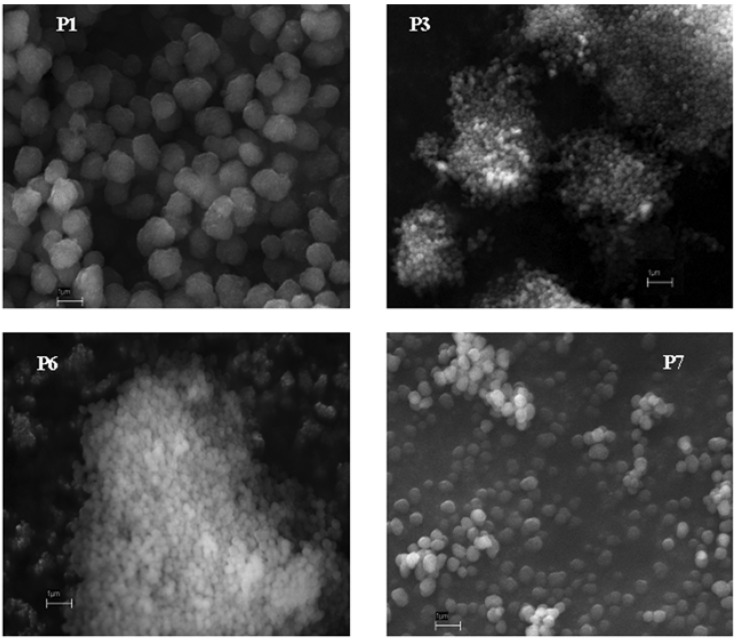
Scanning electron microscopy (SEM) images of P1, P3, P6 and P7 polymers (20,000X).

We have started from a typical experimental precipitation polymerization procedure similar to that described in our previous paper with slight variations. Thus, nia and MAA in molar ratio 1:4 have been dissolved in chloroform and kept into a ultrasonic bath to promote non-covalent interactions to each other. Then, EGDMA (molar ratio MAA/EGDMA ¼) and AIBN have been added and the solution heated at 60°C for 20 h. After removal of the template and after drying the polymer P1 is obtained in about 60% of yield and characterized by SEM. The morphology observed from the SEM image shown in Figure 1 displays spherical particles with very narrow size distribution peaked at about (2 ± 0.20) μm. A non-imprinted polymer (N-P1), prepared following the same procedure described above except for the template, has been also synthesized. N-P1 polymer shows homogenous spherical particles with morphology and yield comparable to that measured for the imprinted polymer P1. Since polymer P1 has been obtained with satisfactory yield and morphology it has been used along with its corresponding non-imprinted polymer N-P1 in rebinding study.

The influence of porogenic solvents on recognition properties as reported in the literature [27,29] was examined neglecting the solvent effect on the polymer synthesis step. In the present work we have studied the solvent effect on yield and morphology size. Acetonitrile, with a higher dielectric constant than chloroform, is a typical porogenic solvent for MIP synthesis and it has been chosen for the synthesis of polymer P2, using the same procedure described for polymer P1. We can observe that the nature of the solvent is critical in the nia-MIPs synthesis step since P2 has been isolated with a very low yield, less than 5%. Thus, we have not taken into consideration this polymer for further characterization.

Successively, different experiments have been performed changing other reaction parameters to understand the influence of these factors on the synthesis efficiency in acetonitrile solvent. When we increased only the temperature from 60 °C to 65 °C the polymer P3 was obtained in higher yield (19 %) than P2, and hence it was characterized by SEM analysis. The homogenous microspheres of polymer P3, shown in Figure 1, appear considerably smaller (0.20 ± 0.02 μm) than the microspheres of the polymer synthesized in chloroform. From these results it seems that polymerization process in acetonitrile needs higher temperature than in chloroform. In polymer P4 the volume of the solvent was reduced from 40 mL (P3) to 30 mL and we found only a slight increase in the microspheres’ size. Increasing the temperature from 65 °C to 70 °C gave a polymer P5 in 43 % of yield. The SEM image of polymer P6 is also shown in Figure 1; in this case the reaction time was increased from 20 h to 48 h, obtaining a performance comparable to that found for the imprinted polymer P5. Therefore, we can conclude that reaction time do not significantly influence the reaction process. Finally, increasing the cross-linker/functional monomer ratio from 1:4 to 1:6, a polymer P7 was obtained with a very high yield and increased size (Figure 1) in comparison to our previous syntheses. This last experiment gave us better results and polymer P7 has been used in the rebinding study. Its non-imprinted polymer N-P7 has been also synthesized. In accordance with chloroform results, also in this case blank polymer (N-P7) is similar to its related imprinted polymer (P7). In conclusion, from this study we can observe that the nature of the solvent is the most important parameter since it completely changes the reaction course; an increasing of temperature or cross-linker amount also gave an improved reaction yield.

### 2.2. Prepolymerization-complexation studies on the interactions between template and functional monomer

Studies on the interactions between template, functional monomer and solvent have been performed by ^1^H-NMR measurements in two different solvents. The nia and MAA molecules have been mixed in CDCl_3_ or MeCN-d_3_ in the same ratio and at similar concentrations as those utilised for polymer synthesis. The chemical shifts of the amide protons of nia in the absence or presence of MAA in CDCl_3_ and MeCN-d_3_ are reported in Table 2.

**Table 2:** ^1^H-NMR chemical shift changes of NH_2_ amide protons of nia with or without MAA in CDCl_3_ or MeCN-d_3_ solvents.

		Chemical shift (δ, ppm)		
Solvent		nia	nia + MAA	Δδ
**CDCl_3_**	H Amide	5.93	6.51	0.58
		6.17	7.00	0.83
**MeCN-*d_3_***	H Amide	6.22	6.60	0.38
		6.90	7.03	0.13

The signals of the amide protons of nia in CDCl_3_ in the absence of MAA appear at 5.93 and 6.17 ppm. The signals shift downfield when MAA is added to the solution (Δδ, 0.58 and 0.83 ppm). In contrast, all pyridinic protons were minimally shifted by addition of MAA. Moreover, no additional proton signals are present, so there is no evidence of hydrogen transfer with ionic compound formation ascribable to the protonation of the nia amide nitrogen site. We can argue that the interaction between acid and base is mainly based on hydrogen-bonding between amide protons and MAA. A similar behaviour has been observed in MeCN-d_3_ solvent, where downfield shifts of the nia amide protons of 0.38 and 0.13 ppm have been measured. The amide protons are less shifted in MeCN-d_3_ than in CDCl_3_, suggesting that the interaction between functional monomer and template is weaker in MeCN.

The signals of the amide proton of nia in CDCl_3_ in the absence of MAA appear at 5.93 and 6.17 ppm. When the same experiment has been performed in MeCN-d_3_ the amide signals occur downfield shifted at 6.22 and 6.90 ppm. These results agree well with the hydrogen bonding–acceptor capacity of the MeCN solvent. Hydrogen bonding certainly occurred between nia and MeCN, probably this interaction makes less strong hydrogen bonding between nia and MAA.

### 2.3. Computational Studies on the Complexes Between nia and MAA

In this section the results of the theoretical studies on the MAA– nia complexes are shown. First the chosen level of theory for the discussed binding energies (RI-BLYP/SV(P) for the structure optimization and B3LYP/TZVP single point calculations for the binding energies) was validated by a comparison with values obtained from more computationally-intensive methods. A reoptimization of the 1:1 and the 1:2 complexes of the template with MAA on the RI-BLYP/TZVP niveau showed no significant changes (compare Table 3 the first two lines for an example). This leads to the conclusion that the RI-BLYP/SV(P) approach is sufficient for the structural optimization. The influence of the basis set superposition error (BSSE) was also estimated by the counterpoise (CP) correction [48] for the 1:1 and the most stable 1:2 complexes (Table 3 lines 3 and 4). With the applied triple zeta basis set this error is about 0.5 kcal/mol for each hydrogen bond (computed on the B3LYP/TZVP//RI-BLYP/SV(P) level of theory).

**Table 3:** A comparison of the obtained binding energies between various theoretical approaches for two selected 1:1 complexes and the most stable 1:2 complex between nia and MAA.

Approach	3	4	1:2
RI-BLYP/def2-TZVP//RI-BLYP/SV(P)	-4.2	-13.9	-24.4
RI-BLYP/def2-TZVP//RI-BLYP/def2-TZVP	-4.4	-14.0	-24.5
B3LYP/def2-TZVP//RI-BLYP/SV(P)	-4.9	-14.7	-25.9
B3LYP/def2-TZVP//RI-BLYP/SV(P) with BSSE	-4.5	-14.1	-24.7
RI-MP2/def2-TZVPP//RI-BLYP/SV(P)	-7.0	-15.9	-29.0

To validate the B3LYP/TZVP//RI-BLYP/SV(P) approach the binding energies were also computed on the RI-MP2/TZVPP//RIBLYP/SV(P) level of theory [55] (Table 3 last line). These energies are about 2 kcal/mol lower for each H-bond, but show the same trends. This leads to the conclusion that the B3LYP/TZVP//RI-BLYP/SV(P) level is a suitable approach for the computation of binding energies in MAA–nia complexes.

**Figure 2:**
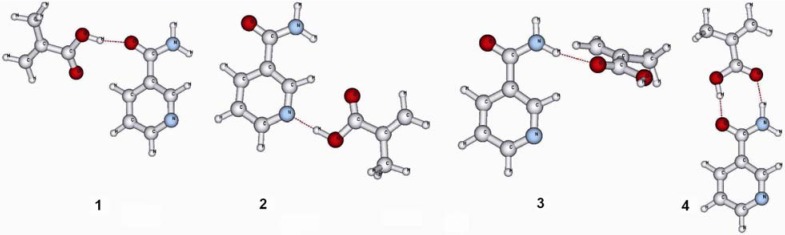
Schematic representation of all possible 1:1 complexes between nia template and MAA functional monomer.

Figure 2 shows the 1:1 complexes at all four possible binding sites of nia. The computed binding energies are summarized in Table 4. In the first column the binding energies as they are computed in the gas phase are given. The second column lists the binding energies in an environment with the dielectric constant of chloroform while the third column shows the binding energies in acetonitrile. The values in parenthesis are the BSSE corrected values. For the stabilization energies computed in a polar surrounding the CP correction obtained from the gas phase computations was used.

From the computed values for the binding energies of the 1:1 complexes (compare Table 4) it can be seen that there is a strong difference in the strength of the hydrogen bonds between MAA and nia at the four binding sites. The double hydrogen bond between the amide group of nia and the carboxyl group of MAA (**4**) gives the best stabilization in gas phase (14.7 kcal/mol). In contrast, the hydrogen bond between an amidic proton of nia and the carbonyl oxygen of MAA (**3**) provides only a weak stabilization of 4.9 kcal/mol. The well known trend that the binding energy of a hydrogen bond is decreased when going from the gas phase to a polar surrounding can be seen for all four possible hydrogen bonds. In the more polar acetonitrile surrounding the hydrogen bond between the amidic proton of nia and the carbonyl oxygen of MAA (**3**) does not provide any significant stabilization.

**Figure 3:**
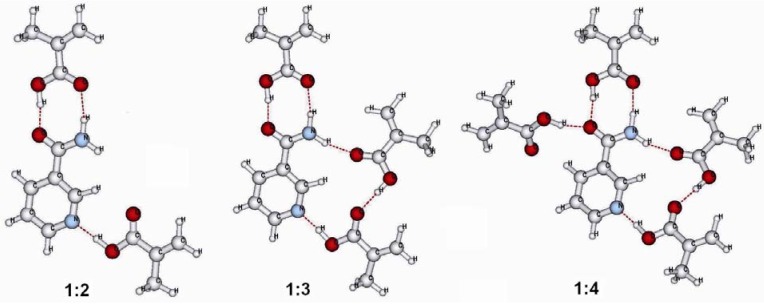
The most stable prepolymerization-complex structures for a ratio of 1:2, 1:3 and 1:4 between nia template and MAA functional monomer.

The experimental preparation of the molecularly imprinted MAA polymer with nia provides an excess of MAA affording to each template molecule the possibility to bind up to four functional monomers in the prepolymerization-complexation step. Figure 3 shows the most stable complexes between nia and MAA for a ratio of 1:2, 1:3 and 1:4 between template and functional monomer. The computed stabilization energies are given in Table 5.

**Table 4:** Computed binding energies (in kcal/mol with and without BSSE correction) of all possible 1:1 complexes between nia and MAA in gas phase and in different polar surroundings.

Hydrogen bond	Gas phase	CHCl_3_ (ε = 4.81)	MeCN (ε = 37.5)
**1**	-9.2 (-8.7)	-5.4 (-4.9)	-4.2 (-3.8)
**2**	-11.5 (10.9)	-7.4 (-6.8)	-5.8 (-5.2)
**3**	-4.9 (-4.5)	-2.1 (-1.7)	-0.8 (-0.4)
**4**	-14.7 (14.1)	-9.1 (-9.5)	-6.7 (-6.1)

**Table 5:** The computed binding energies (in kcal/mol) of the prepolymerization-complexation between nia and MAA shown in Figure 3 in gas phase and in the different polar surroundings.

Surrounding	1:2	1:3	1:4
**Gas phase**	-25.9	-38.5	-46.2
**CHCl_3_ (ε = 4.81)**	-16.1	-23.0	-27.1
**MeCN (ε = 37.5)**	-12.3	-16.7	-19.9

From the values for the stabilization energies between nia and more than one template molecule summarized in Table 5 it can be seen that the addition of each new MAA molecule to the complex provides an additional stabilization. This effect is very strong in the gas phase, but is also found in the polar surroundings of chloroform and acetonitrile. From the structures shown in Figure 3 it can be seen that a framework of hydrogen bonds is also formed between the functional monomer molecules. This leads to a good stabilization of the prepolymerization-complex. This also should help to form good cavities in the MIP after extracting the nia molecules from the polymer. A good rebinding property of the polymer to the nia template can then be expected from this prepolymerization-complexation behaviour.

**Figure 4:**
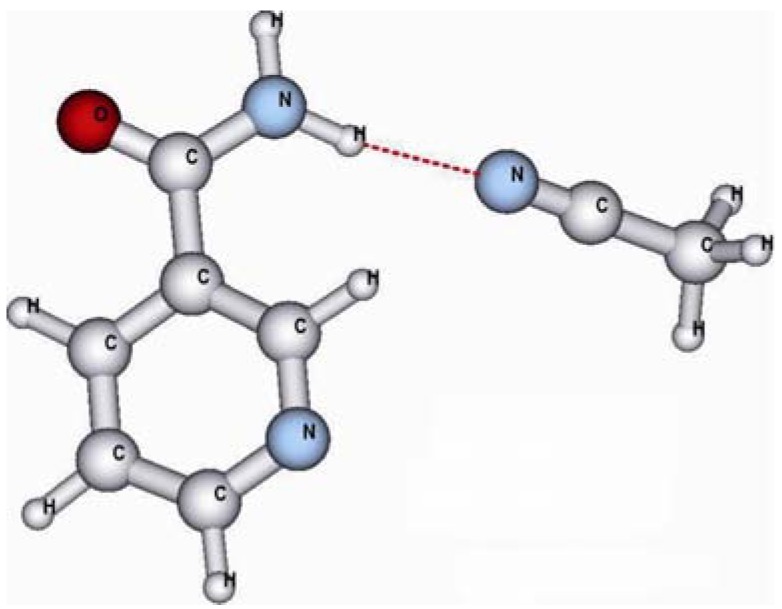
The hydrogen bond between MeCN and nia.

Since the acetonitrile solvent molecules also have a hydrogen bond acceptor capacity it is possible that a MeCN molecule forms a hydrogen bond to the amidic protons if MeCN is used as polymerization porogen solvent. This is indeed the case as the ^1^H-NMR studies indicate. To verify this hypothesis the hydrogen bond complex between an acetonitrile molecule and the amidic proton of nia is also computed (cf. Figure 4).

A comparison of the computed stabilization energies (Table 6) between the hydrogen bond to the MeCN solvent molecule and the hydrogen bond formed between the template molecule and the amidic proton of nia shows, that the solvent molecules are able to build stronger hydrogen bonds at this position than MAA. Since the computed energy differences are very small this finding is additionally checked with the RI-MP2 approach.

**Table 6:** The binding energy (in kcal/mol) of the hydrogen bond of **3** and the hydrogen bond between nia and MeCN for various theoretical approaches in comparison.

Approach	nia-MAA	nia-MeCN
B3LYP/def2-TZVP (gas phase)	-4.9	-5.4
B3LYP/def2-TZVP with BSSE (gas phase)	-4.5	-5.2
B3LYP/def2-TZVP (MeCN ε = 37.5)	-0.8	-1.2
B3LYP/def2-TZVP (MeCN ε = 37.5) + BSSE	-0.4	-1.0
RI-MP2/def2-TZVPP (gas phase)	-7.0	-7.1
RI-MP2/def2-TZVPP with BSSE (gas phase)	-5.9	-6.6

For all levels of theory the hydrogen bond of nia to the solvent is computed to give a bigger stabilization. This effect is even stronger if the BSSE correction is included in the theoretical values. This interaction with the solvent blocks the amidic proton from the binding to the functional monomer, and the framework of hydrogen bonds between the MAA molecules can not be established. As a consequence an effective complexation of nia in acetonitrile as polymerization porogen solvent is not possible. Since this effective prepolymerization-complexation is necessary for a good imprinting process it can be expected that the MIP prepared in MeCN as polymerization porogen solvent shows a bad recognition behavior towards nia. This is found in the experiment for the polymer P7 which was synthesized in acetonitrile as polymerization porogen solvent.

### 2.4. Binding Capacity Evaluation

**Figure 5:**
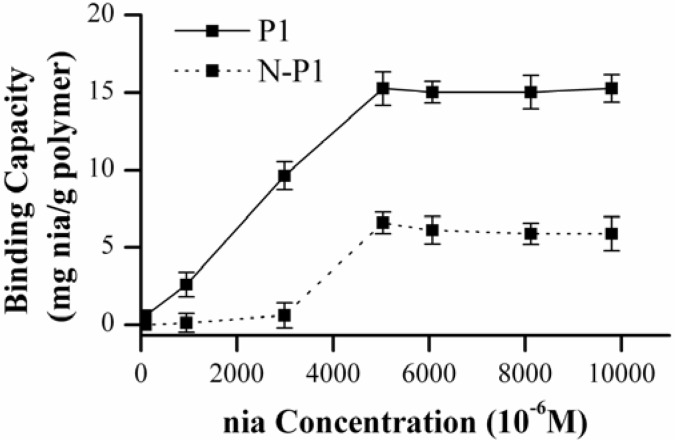
Binding capacity in CHCl_3_ of imprinted polymer P1 and non-imprinted polymer N-P1 versus nia concentration.

Binding capacity of P1 and P7 synthesized in chloroform and MeCN, respectively, has been evaluated by re-binding studies. Their non-imprinted polymers N-P1 and N-P7 have been also used as a reference. The binding capacity in CHCl_3_ of imprinted polymer P1 and non-imprinted polymer N-P1 versus nia concentration has been reported in Figure 5. 

**Figure 6:**
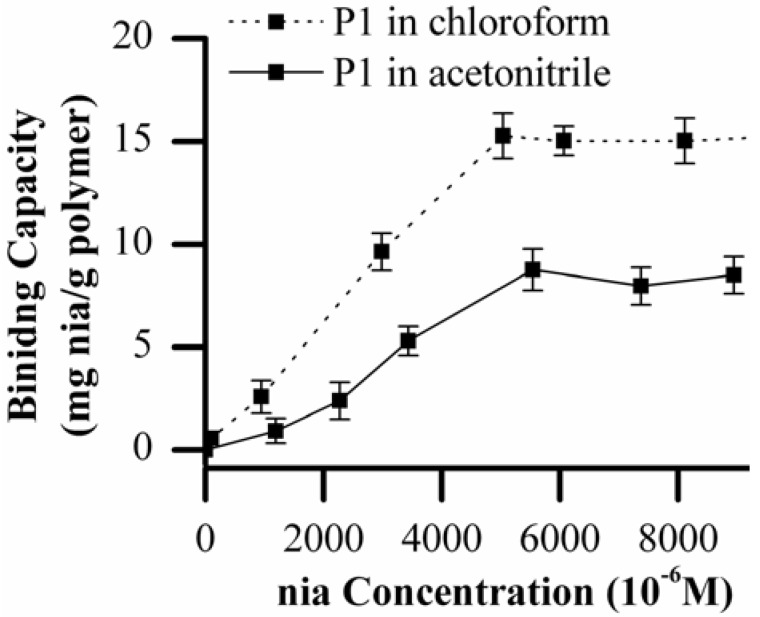
Binding capacity of imprinted polymer P1 in CHCl_3_ and MeCN versus nia concentration.

The imprinted polymer P1 curve shows a typical trend, the amount of nia re-bound to the polymer increases with the increasing initial concentration of nia from 0.1 × 10^-3^ M to 5 × 10^-3^ M reaching a saturation plateau at higher concentrations. As expected, non-imprinted polymer N-P1 shows a less sensitive response to the template. This confirms that during the polymer synthesis a reasonable number of specific binding sites are formed and the polymer P1 has a good affinity for nia template. The affinity of MIPs is affected by solvents with different hydrogen-bonding capacities used in the re-binding experiments. So that, the binding capacity evaluation of MIP P1 has been performed using MeCN as solvent. In Figure 6 these results have been compared with the previous curve obtained in chloroform. MeCN as re-binding solvent shows a weaker imprint effect than chloroform. MeCN unlike the chloroform enables to make hydrogen bonding with nia interfering with nia availability in the re-binding process.

The solvent influence on the interaction between template and MAA and the formation of specific recognition sites in imprinting process has been considered by using polymer P7, synthesized in MeCN, in the re-binding studies. Unlike polymer P1 results, polymer P7 has shown no significant binding capacity when chloroform or MeCN are used as the incubation solvents. Probably MeCN interfered with hydrogen bonding between template and MAA during the polymerization step of the polymer P7. So that, the formation of poor recognition site occurred when MeCN is used as polymerization porogen solvent. This agrees well with ^1^H NMR assumption.

### 2.5. Selectivity Evaluation

**Figure 7:**
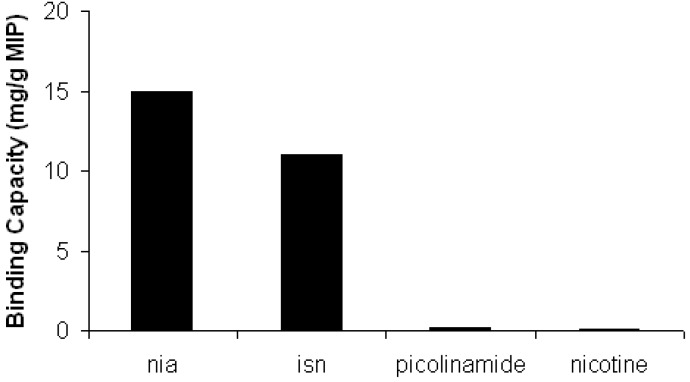
Binding capacity of imprinted polymer P1 versus different nitrogen bases.

The different affinity of imprinted polymer P1 towards other three similar nitrogen bases, such as isonicotinamide (isn), picolinamide and nicotine, has been investigated. 5 × 10^-3^ M chloroform solution of picolinamide, isn and nicotine have been incubated with imprinted polymer P1 using the same procedure employed with nia. Each calculated binding capacity has been shown in Figure 7. Picolinamide and isn are two positional isomers of nia that have the amide group linked to a different pyridinic ring carbon. The greatest results are obtained for nia, but also isn shows a significant affinity towards imprinted polymer P1, but lower than nia. Picolinamide, unlike isn, did not show any affinity towards imprinted polymer P1 even though it is structurally similar to nia. This difference can be ascribed to the intramolecular hydrogen bond in picolinamide compound. Finally, nicotine with a different structure did not show any affinity towards imprinted polymer P1.

## 3. Conclusions

We have reported in this work for the first time the preparation of imprinted microspheres in presence of nia as print molecule using a precipitation polymerization method. We have studied the influence of some parameters in order to optimise the experimental conditions and from this study we can observe that the nature of the solvent is the most important parameter, since it completely changes the reaction course; furthermore an increase in temperature or an increase of the amount of cross-linker improves the yields of the reaction.

Complexation studies on possible interactions between template and functional monomer were also performed in chloroform and MeCN using ^1^H-NMR. We have found that the interaction between nia and MAA in both solvents studied is mainly based on hydrogen-bonding. The theoretical results show a framework of hydrogen bonds that is also established between the functional monomer molecules. Moreover, computational results (H-bond stabilisation energies for the bonds between the template and the functional monomer or the nia molecule and the solvent) and experimental results (^1^H NMR shift changes of the amidic protons) agree that the interaction between nia and MAA is less strong in MeCN, for the presence of hydrogen bonding occurred between nia and MeCN. These results are in line with the studies carried out by Prachayasittikul and coworkers [53,54] suggesting that computational studies can be helpful for a preliminary assessment of template, functional monomer, solvent interactions in MIPs studies.

The binding ability of MIP systems towards different concentration of nia solutions in various solvent systems has been evaluated by spectrophotometric analysis. The imprinted activity of polymer P1, synthesized in chloroform, has been demonstrated when chloroform or MeCN are used as incubation solvents. Whereas, polymer P7, synthesized in MeCN, has shown no significant binding capacity in both solvents. Probably MeCN interfered with hydrogen bonding between template and MAA during the polymerization step and re-binding process.

The selectivity of P1 towards nia by considering other analogous compounds, such as nicotine and its isomers isonicotinamide (isn) and picolinamide has been also confirmed.

## 4. Experimental

### 4.1. General

The compounds nia (99+ %), isn (≥ 99 %), picolinamide (> 98 %), nicotine (> 99 %, GC), methacrylic acid (> 99 %), ethylene glycol dimethacrylate (> 98 %), azobis(isobutyronitrile) (AIBN, > 98 %), were purchased from Aldrich and used as received. Ultrapure water was obtained from a model New Human Power I ultrapure water system from Human Corporation. Acetonitrile (MeCN) (Baker, analyzed grade) was dried by leaving overnight over molecular sieves and then distilled from calcium hydride before use. Dry chloroform (Aldrich, ≥ 99.8 % A.C.S. reagent) was passed through a column of basic alumina (Aldrich, standard grade) before use. All other solvents (Baker, analyzed grade) were used without further purification.

Sonication was carried out using a Sonorex RK 102H ultrasonic water bath from Bandelin Electronic. Centrifugation was achieved with a PK121 multispeed centrifuge from Thermo Electron Corporation. A Heidolph Instrument Rotamax 120 type rocking table was used for shaking incubated mixtures. ^1^H-NMR spectra were recorded on a Bruker Avance 400 NMR spectrometer at room temperature and chemical shifts were reported relative to tetramethylsilane. Absorbances were measured by UV Vis spectrophotometer Cary 100 scan (Varian). The morphology of the microspheres was analyzed by using a ZEISS EVO 40 scanning electron microscopy (SEM) in high vacuum mode without prior treatment.

### 4.2. Polymers Preparation and Template Removal

Syntheses of nia imprinted microspheres were carried out following the method described in our previous paper [52] with slight variations. The conditions used for the synthesis are summarized in Table 1: in every experiment, 0.43 mmol of nia and 1.55 mmol of MAA were dissolved in the solvent in a 100 mL three necked round bottom flask and the solution was kept into a ultrasonic bath for 10 min; then, EGDMA and 0.15 mmol of AIBN were added to the solution. The reaction mixture was saturated with nitrogen for 10 min and heated for 20 h or 48 h to allow polymerization. After cooling at room temperature, the mixture was sonicated for further 5 min and the microspheres separated by filtration. The template in the microspheres was removed by washing several times with 20 ml of ethanol/acetic acid (8/2 v/v) solution until nia signal at 262 nm was not detected and then washed with ethanol in order to remove the acetic acid present. Finally the microspheres were dried and stored under vacuum to avoid any contamination. As a control, non-imprinted microspheres (NIP) were also prepared following the same procedure above described except for the template.

### 4.3. Computational Details

**Figure 8:**
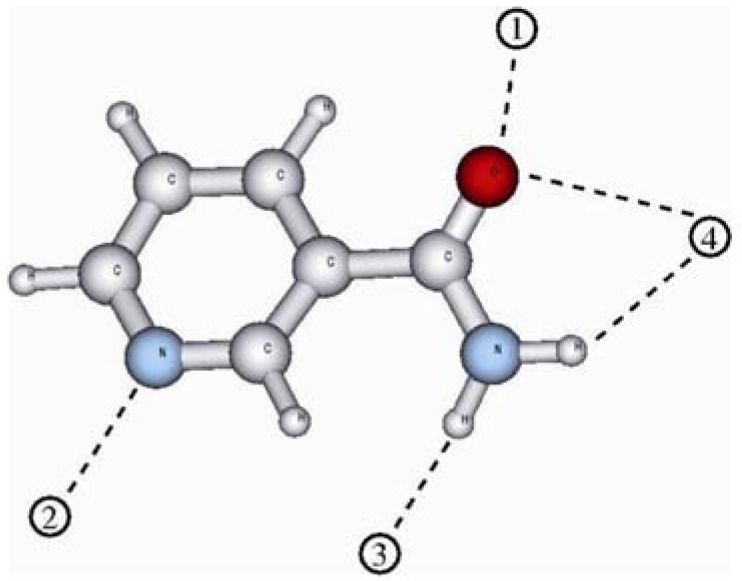
The possible binding sites for the functional monomer MAA to the nicotinamide template molecule.

To study the binding situation in the prepolymerization-complexation step, all possible combinations of nia with the functional monomer MAA (see Figure 8) were build up with the aid of the AVOGADRO program [56] and preoptimized by the MMFF94 [57] forcefield applying its implementation in the avogadro program. The obtained structures were then fully optimized using density functional theory (DFT) on the BLYP/SV(P) niveau [41,42,58] applying the TURBOMOLE program package [59] . For these calculations the resolution of identity (RI) approximation [34,35] together with the corresponding auxiliary basis sets [35,60] provided by TURBOMOLE were applied. The binding energies then were obtained by single point calculations on the B3LYP/TZVP//RI-BLYP/SV(P) niveau [42,43,61] according to:

Binding energy = (Energy of nia-MAA complex) - (Energy of nia) - x × (Energy of MAA)

where x stands for the amount of MAA molecules in the complex.

The MAA–nicotinamide complexes were optimized in gas phase and in a polar environment. This was done with the COSMO [62] approach for chloroform (ε = 4.81) and acetonitrile (ε = 37.5). The influence of the basis set superposition error (BSSE) was also estimated by the counterpoise (CP) correction [48]. The applied level of theory was validated by a comparison of the computed binding energies with values obtained from RI-BLYP/TZVP//RI-BLYP/TZVP and RI-MP2/TZVPP// RIBLYP/SV(P) computations [55] (section Results and Discussion, Table 3).

### 4.4. Calibration Curves and Binding Experiments

To evaluate the amount of template extracted during the washing step calibration curves were prepared reporting absorbance versus template concentration. Similarly calibration curves were achieved when template or its analogues were used in the re-binding stages.

In a polypropylene tube, MIP or NIP (20 mg) were suspended in MeCN (5.0 mL) containing nia at a known concentration ranging from 1 × 10^-4^ M to 1 × 10^-2^ M. The mixture was incubated for 16 h using a rocking table working at room temperature and 75 rpm. After centrifugation at 8,000 rpm for 10 min, the supernatant was filtered through a 0.20 µm porosity polypropylene filter. The nia concentration in the solution after the binding process was determined by measuring the absorbance at 262 nm and the result was compared with concentration before incubation. Re-binding experiments were also performed in chloroform solutions following the same procedure except for the use of glass tube. Analogously to nia, solutions of nicotine, isonicotinamide or picolinamide at the concentration of 5 × 10^-3^ M in chloroform were incubated with polymer microspheres and treated as above reported. Nicotine, isonicotinamide or picolinamide concentrations in the solution after the binding process were determined by measuring the absorbance at 262 nm, 268 nm and 265 nm respectively and compared with their initial concentration. Binding processes and measurements were performed in triplicates and their average binding percentages were calculated.

## References

[1] Sellergren B. (1994). Direct drug determination by selective sample enrichment on an imprinted polymer. Anal. Chem..

[2] Sellergren B. (1999). Polymer- and template-related factors influencing the efficiency in molecularly imprinted solid-phase extractions. Trends Anal. Chem..

[3] Haginaka J. (2004). Molecularly imprinted polymers for solid-phase extraction. Anal. Bioanal. Chem..

[4] Koster E.H.M., Crescenzi C., den Hoedt W., Ensing K., de Jong G.J. (2001). Fibers coated with molecularly imprinted polymers for solid-phase microextraction. Anal. Chem..

[5] Takeuchi T., Haginaka J. (1999). Separation and sensing based on molecular recognition using molecularly imprinted polymers. J. Chromatogr. B.

[6] Kempe M., Mosbach K. (1995). Molecular imprinting used for chiral separations. J. Chromatogr..

[7] Sellergren B. (2001). Imprinted chiral stationary phases in high-performance liquid chromatography. J. Chromatogr..

[8] Kriz D., Ramstrom O., Mosbach K. (1997). Molecular imprinting. New possibilities for sensor technology. Anal. Chem..

[9] Henry O.Y.F., Cullen D.C., Piletsky S.A. (2005). Optical interrogation of molecularly imprinted polymers and development of MIP sensors: a review. Anal. Bioanal. Chem..

[10] Haupt K., Mosbach K. (2000). Molecularly Imprinted Polymers and Their Use in Biomimetic Sensors. Chem. Rev..

[11] Tada M., Iwasawa Y. (2003). Approaches to design of active structures by attaching and molecular imprinting of metal complexes on oxide surfaces. J. Mol. Catal. A: Chem..

[12] Wulff G., Diederich F., Stang P.J. (2000). Templated Synthesis of Polymers - Molecularly Imprinted Materials for Recognition and Catalysis. Templated Organic Synthesis.

[13] Alvarez-Lorenzo C., Concheiro A. (2004). Molecularly imprinted polymers for drug delivery. J. Chromatogr. B.

[14] Hilt J.Z., Byrne M.E. (2004). Configurational biomimesis in drug delivery: molecular imprinting of biologically significant molecules. Adv. Drug Deliv. Rev..

[15] Wulff G., Poll H.G. (1987). Enzyme-analog built polymers. 23. Influence of the structure of the binding sites on the selectivity for racemic resolution. Makromol. Chem..

[16] Wulff G., Yan M., Ramstroem O. (2005). The Covalent and Other Stoichiometric Approaches. Molecularly Imprinted Materials.

[17] Arshady R., Mosbach K. (1981). Synthesis of substrate-selective polymers by host-guest polymerization. Macromol. Chem. Phys..

[18] Mosbach K., Ramstrom O. (1996). The emerging technique of molecular imprinting and its future impact on biotechnology. Bio/Technology.

[19] Mayes A.G., Mosbach K. (1996). Molecularly imprinted polymer beads: suspension polymerization using a liquid perfluorocarbon as the dispersing phase. Anal. Chem..

[20] Lee S.W., Yang D.H., Kunitake T. (2005). Regioselective imprinting of anthracenecarboxylic acids onto TiO_2_ gel ultrathin films: an approach to thin film sensor. Sens. Actuat. B.

[21] Li K., Stover H.D.H. (1993). Synthesis of monodisperse poly(divinylbenzene) microspheres. J. Polym. Sci. Part A: Polym. Chem..

[22] Turiel E., Martin-Esteban A. (2004). Molecularly imprinted polymers: towards highly selective stationary phases in liquid chromatography and capillary electrophoresis. Anal. Bioanal. Chem..

[23] Wang J., Cormack P.A.G., Sherrington D.C., Khoshdel E. (2003). Monodisperse, molecularly imprinted polymer microspheres prepared by precipitation polymerization for affinity separation applications. Angew. Chem. Int. Ed..

[24] Ye L., Weiss R., Mosbach K. (2000). Synthesis and characterization of molecularly imprinted microspheres. Macromolecules.

[25] Ye L., Cormack P.A.G., Mosbach K. (2001). Molecular imprinting on microgel spheres. Anal. Chim. Acta.

[26] Williams A., Ramsden D. (2005). Nicotinamide: A double edged sword. Parkinsonism Relat. Disorders.

[27] Fu Q., Zheng N., Li Y.Z., Chang W.B., Wang Z.M. (2001). Molecularly imprinted polymers from nicotinamide and its positional isomer. J. Mol. Recognit..

[28] Wu L., Li Y. (2004). Study on the recognition of templates and their analogues on molecularly imprinted polymer using computational and conformational analysis approaches. J. Mol. Recognit..

[29] Wu L., Zhu K., Zhao M., Li Y. (2005). Study on the recognition of templates and their analogues on molecularly imprinted polymer using computational and conformational analysis approaches. Anal. Chim. Acta..

[30] Li Z., Yang G., Liu S., Chen Y. (2005). Adsorption isotherms on nicotinamide-imprinted polymer stationary phase. J. Chromatogr. Sci..

[31] Zhang Z., Li H., Liao H., Nie L., Yao S. (2005). Influence of cross-linkers amount on the performance of the piezoelectricsensor modified with molecularly imprinted polymers. Sens. Actuat. B.

[32] Dethlefs K.M., Hobza P. (2000). Ncovalent interactions: a challenge for experimental and theory. Chem. Rev..

[33] Hohenberg P., Kohn W. (1964). Inhomogeneous Electron Gas. Phys. Rev..

[34] Kohn W., Sham L.J. (1965). Quantum density oscillations in an inhomogeneous electron gas. Phys. Rev..

[35] Vahtras O., Almlöf J., Feyereisen W. (1993). Integral approximations for LCAO-SCF calculations. Chem. Phys. Lett..

[36] Eichkorn K., Treutler O., Öhm H., Häser M., Ahlrichs R. (1995). Auxiliary basis sets to approximate Coulomb potentials. (Chem. Phys. Letters 240 (1995) 283). Chem. Phys. Lett..

[37] Tsuzuki S., Lüthi H.P. (2001). Interaction energies of van der Waals and hydrogen bonded systems calculated using density functional theory: assessing the PW91 model. J. Chem. Phys..

[38] Jurečka P., Černý J., Hopza P., Salahub D.R. (2007). Density functional theory augmented with an empirical dispersion term. Interaction energies and geometries of 80 noncovalent complexes compared with Ab Initio quantum mechanics calculations. J. Comp. Chem..

[39] Elstner M., Hobza P., Frauenheim T., Suhai S., Kaxiras E. (2001). Hydrogen bonding and stacking interactions of nucleic acid base pairs: a density-functional-theory based treatment. J. Chem. Phys..

[40] Tuma C., Boese A.D., Handy N.C. (1999). Predicting the binding energies of H-bonded complexes: a comparative DFT study. Phys. Chem. Chem. Phys..

[41] Becke A.D. (1988). Density-functional exchange-energy approximation with correct asymptotic behavior. Phys. Rev. A.

[42] Lee C., Yang W., Parr R.G. (1988). Development of the Colle-Salvetti correlation-energy formula into a functional of the electron density. Phys. Rev. B.

[43] Becke A.D. (1993). Density-functional thermochemistry. III. The role of exact exchange. J. Chem. Phys..

[44] Perdew J.P., Ernzerhof M., Burke K. (1996). Rationale for mixing exact exchange with density functional approximations. J. Chem. Phys..

[45] Perdew J.P., Burke K., Ernzerhof M. (1996). Generalized gradient approximation made simple. Phys. Rev. Lett..

[46] Ireta J., Neugebauer J., Scheffler M. (2004). On the accuracy of DFT for describing hydrogen bonds: dependence on the bond directionality. J. Phys. Chem. A.

[47] Long Q., Ji H., Lv S. (2009). DFT study on the hydrogen bonds of phenol–cyclohexanone and phenol–H_2_O_2_ in the Baeyer–Villiger oxidation. Int. J. Quant. Chem..

[48] Boys S.F., Bernardi F. (1970). The calculation of small molecular interactions by the differences of separate total energies. Some procedures with reduced errors. Mol. Phys..

[49] Novoa J.J., Sosa C. (1995). Evaluation of the density functional approximation on the computation of hydrogen bond interactions. J. Phys. Chem..

[50] Hobza P., Šponer J., Reschel T. (1995). Density functional theory and molecular clusters. J. Comp. Chem..

[51] Tomura M. (2008). Theoretical study for a complex of 1,2,5-thiadiazole with formic acid. J. Mol. Struct. (Theochem).

[52] Del Sole R., De Luca A., Catalano M., Mele G., Vasapollo G. (2007). Noncovalent imprinted microspheres: preparation, evaluation and selectivity of DBU template. J. Appl. Polym. Sci..

[53] Nantasenamat C., Isarankura-Na-Ayudhya C., Bulow L., Ye L., Prachayasittikul V. (2006). In silico design for synthesis of molecularly imprinted microspheres specific towards bisphenol A by precipitation polymerization. EXCLI J..

[54] Isarankura-Na-Ayudhya C., Nantasenamat C., Buraparuangsang P., Piacham T., Ye L., Bulow L., Prachayasittikul V. (2008). Computational insights on sulfonamide imprinted polymers. Molecules.

[55] Weigend F., Hätting C., Köhn A. (2002). Efficient use of the correlation consistent basis sets in resolution of the identity MP2 calculations. J. Chem. Phys..

[56] http://avogadro.openmolecules.net/

[57] Halgren T.A. (1996). Merck molecular force field. I. Basis, form, scope, parameterization, and performance of MMFF94. J. Comp. Chem..

[58] Schäfer A., Horn H., Ahlrichs R. (1992). Fully optimized contracted Gaussian basis sets for atoms lithium to Krypton. J. Chem. Phys..

[59] Ahlrichs R., Bär M., Baron H.P., Bauernschmitt R., Böcker S., Ehrig M., Eichkorn K., Elliott S., Haase F., Häser M., Horn H., Huber C., Huniar U., Kattannek M., Kölmel C., Kollwitz M., Ochsenfeld C., Öhm H., Schäfer A., Schneider U., Treutler O., von Arnim M., Weigend F., Weis P., Weiss H. TURBOMOLE.

[60] Eichkorn K., Treutler O., Öhm H., Häser M., Ahlrichs R. (1995). Auxiliary basis sets to approximate Coulomb potentials. Chem. Phys. Lett..

[61] Schäfer A., Huber C., Ahlrichs R. (1994). Fully optimized contracted Gaussian basis sets of triple zeta valence quality for atoms Li to Kr. J. Chem. Phys..

[62] Klamt A., Schüurmann G. (1993). COSMO: A new approach to dielectric screening in solvents with explicit expressions for the screening energy and its gradient. J. Chem. Soc. Perkin Trans. 2.

